# Steam Explosion as a Green Pretreatment Strategy to Enhance Total Phenolics Release and Biological Activities in *Potentilla discolor* Bunge Stems

**DOI:** 10.3390/molecules31010139

**Published:** 2025-12-31

**Authors:** Xiao Zhang, Yuchen Cui, Wenjie Sui, Mengqi Cheng, Xinyu Xu, Wanting Duan, Ziyi Cheng, Jiajia Fu, Yanmei Xu, Youxin Li

**Affiliations:** 1 Tianjin Key Laboratory for Modern Drug Delivery and High-Efficiency, Collaborative Innovation Center of Chemical Science and Engineering, School of Pharmaceutical Science and Technology, Faculty of Medicine, Tianjin University, Tianjin 300072, China; 2 State Key Laboratory of Food Nutrition and Safety, Tianjin University of Science & Technology, Tianjin 300457, China; 3 Hebei Institute for Drug and Medical Device Control, Shijiazhuang 050033, China; 4 Neurocritical Care Medicine Innovation Center, Ministry of Education, Tianjin University, Tianjin 300072, China; 5 State Key Laboratory of Advanced Medical Materials and Devices, Tianjin University, Tianjin 300072, China

**Keywords:** *Potentilla discolor* Bunge, steam explosion, total phenolics, antioxidant activity, hypoglycemic activity

## Abstract

To efficiently, sustainably, and rapidly extract bioactive compounds, steam explosion (SE) technology was tried to use for the first time as a pretreatment of *Potentilla discolor* Bunge (PDB) stems. This study systematically investigated the effects of SE on stem structure, total phenolic content, composition and bioactivities. Macroscopic observation showed that SE-treated stems became darker and deeper in color. Microscopic analysis indicated a reduction in hemicellulose and lignin contents, while the basic skeletal structure remained intact, which facilitated the release of active compounds. This structural modification was directly linked to an enhancement in biological activity. Compared with the untreated group, total phenolic content in SE-treated stems increased 1.11–1.94 times. Correspondingly, antioxidant and hypoglycemic activities were enhanced by 1.35–7.19 times, demonstrating a clear relationship between the structural changes and the improved bioactivity. HPLC analysis showed specific changes in chemical composition, with increased levels of total phenolic content, particularly phenolic acids and flavonoids. Compositional analysis using Q Exactive HF LC-MS and standard comparison revealed that complex macromolecules, such as flavonoid glycosides and polyphenols, were hydrolyzed into smaller, more bioavailable molecules, such as quercetin. Overall, SE pretreatment represents a sustainable and effective approach for improving the extraction of bioactive compounds from PDB stems. These active compounds hold significant potential for applications in functional foods, nutraceuticals, and natural health products, offering an innovative strategy to enhance the bioavailability and bioactivity of plant-derived compounds.

## 1. Introduction

*Potentilla discolor* Bunge (PDB), a member of the Rosaceae family, is a commonly used medicinal herb with reported antioxidant, anti-inflammatory, and hypoglycemic activities [1,2,3]. These effects are primarily attributed to its bioactive constituents, including phenolic acids, flavonoids, triterpenoids, polysaccharides, and tannins, among which total phenolics are considered the key contributors to its pharmacological efficacy [4,5,6]. The stems are the main medicinal part of PDB. However, their dense structure and high lignin and hemicellulose content limit the release of active compounds. Therefore, development of the efficient extraction methods is essential for subsequent pharmacological research and practical applications.

To understand the current research landscape of *Potentilla discolor* Bunge, the representative studies were summarized, which applied different extraction approaches from various plant parts. For example, Ayesheh [7] extracted powdered PDB roots using a methanol–water system followed by ethyl acetate re-extraction, obtaining a proanthocyanidin-rich fraction that exhibited cardioprotective effects. Meng [8] isolated a triterpenoid derivative from an ethanol extract of PDB and demonstrated its ability to inhibit the proliferation, migration, and invasion of lung cancer cells by targeting the FAK and ERK/MAPK pathways. Natalia [9] applied ultrasound-assisted extraction with 80% methanol in root material and observed antithrombotic effects in vivo, while Zhang [10] used 85% ethanol under reflux to extract flavonoids from the whole plant, which attenuated LPS-induced inflammation by modulating NF-κB and AP-1 signaling. Collectively, these studies highlighted the diverse chemical constituents and biological activities about PDB. The differences among studies largely reflected the selection of distinct plant parts, extraction solvents, and research endpoints rather than inconsistencies in PDB’s intrinsic pharmacological properties. Nonetheless, pretreatment and extraction conditions significantly influence the obtained chemical profiles and thus may affect the types of compounds available for subsequent biological evaluation. Therefore, pretreatment and extraction strategies not only influence the yield and composition of bioactive compounds but may also affect which biological activities are observed for PDB extracts.

In traditional plant extraction studies, sample pretreatment methods such as grinding, drying, dewaxing, acid-base treatment, and enzymatic hydrolysis are commonly used to enhance the accessibility of intracellular bioactive compounds. However, each of these methods has their limitations when applied in PDB. Grinding can reduce particle size and increase surface area, but the dense tissues and abundant lignified fibers of PDB make grinding alone insufficient to release intracellular compounds. Drying facilitates handling but may cause degradation of sensitive molecules such as phenolic acids and flavonoids. Dewaxing removes surface lipids, yet the low wax content of PDB limits its benefit while adding extra processing steps. Acid-base treatment can chemically break down the cell wall, but strong acids or bases may induce oxidation, hydroxyl cleavage, or other chemical changes in unstable small molecules, and highly lignified tissues may still resist complete disruption [11]. Enzymatic hydrolysis offers specificity under mild conditions, yet its efficiency is constrained by temperature, pH, enzyme activity, long processing times, potential interactions with polyphenols, and limited penetration into lignified tissues [12]. Taken together, these limitations highlight the need for a novel pretreatment strategy that can efficiently disrupt the cell wall, preserve the stability of active constituents, and be environmentally friendly.

Steam explosion (SE) is a pretreatment technology that applies high-temperature and high-pressure saturated steam for a short duration, followed by rapid decompression [13,14]. Owing to its strong permeability and cell wall disruption ability, SE has been increasingly applied to plant materials in recent years and has shown considerable potential for improving the extraction and bioactivity of phytochemicals. For instance, SE pretreatment has been reported to increase polysaccharide yield in *Ampelopsis grossedentata* while enhancing α-glucosidase inhibitory activity [15], to promote the release of phenolic compounds in adzuki beans with improved antioxidant capacity [16], and to facilitate greater liberation of phenolics and flavonoids from wheat bran [17]. Compared with conventional methods such as ultrasound-assisted extraction, acid-base treatment, and enzymatic hydrolysis, SE offers advantages in extraction efficiency, preservation of heat-sensitive compounds, and enhancement of bioactivity. However, its application for phenolic extraction from PDB stems has not yet been reported, leaving a knowledge gap regarding the efficiency of SE in extracting and preserving bioactive phenolics from this plant. Considering that antioxidant and hypoglycemic activities are closely linked to the prevention of chronic diseases such as diabetes, cardiovascular disorders [4,5,6] and aging-related conditions, and that plant-derived extracts with these activities are of increasing interest in functional foods and natural health products, exploring the effect of SE on the phenolic composition and associated bioactivities of PDB stems could provide new insights into their value-added utilization in the food and healthcare industries.

In theory, SE pretreatment could be suitable for cell wall disruption and promote the release of active ingredients such as phenolic acids and flavonoids. This study aims to try to employ SE technology to pretreat PDB stems for efficient component extraction and increasing bioactivity. The effects of different SE pressures and durations on the pretreatment of PDB stems was investigated. The structural changes before and after the pretreatment were characterized by scanning electron microscopy (SEM), fourier transform infrared spectroscopy (FT-IR), thermogravimetric analysis (TGA), and X-ray diffraction (XRD). Their total phenolic contents were determined using the Folin Ciocalteu method. Antioxidant and hypoglycemic activities of the extracts also evaluated. Finally, their chemical compositions were analyzed by high performance liquid chromatography (HPLC) and Quadrupole-Orbitrap exactive high field liquid chromatography–mass spectrometry (Q Exactive HF LC-MS).

## 2. Results and Discussion

### 2.1. Characterization of PDB Stems

To promote the release of bioactive compounds from PDB stems, SE pretreatment was employed. Three pressure levels (0.6 MPa, 1.0 Mpa, and 1.4 MPa) and two treatment durations (6 min and 10 min) were designed. To better understand the changes in the microstructure and physicochemical properties of the plant tissue, different treated PDB stems were systematically characterized using scanning electron microscopy (SEM), fourier transform infrared spectroscopy (FT-IR), thermogravimetric analysis (TGA), and X-ray diffraction (XRD).

#### 2.1.1. Appearance and Structure of PDB Stems Before and After SE Treatment

**Figure 1:**
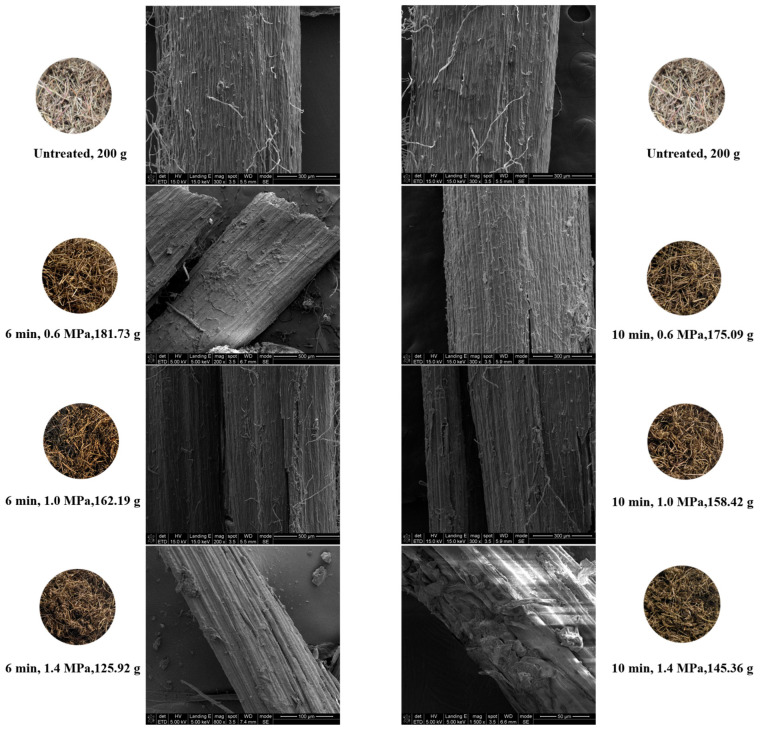
SEM images of PDB stems under different SE conditions.

From the macroscopic perspectives shown in Figure 1, the significant morphological changes were observed in the SE treated PDB stems. As the pressure increased from 0.6 MPa to 1.0 MPa, and eventually to 1.4 MPa, the stems gradually became curled, losing their rigidity, and their color darkened. These changes became more pronounced with longer treatment times. Such phenomena may be related to the elevated temperature and pressure during the SE process, which, according to previous studies, could enhance the interactions between amino compounds and reducing sugars in PDB and thus possibly contribute to Maillard-type reactions [18,19,20]. Correspondingly, the mass of the stems decreased under these conditions: 181.73 g and 175.09 g at 0.6 MPa for 6 min and 10 min, 162.19 g and 158.42 g at 1.0 MPa, and 125.92 g and 145.36 g at 1.4 MPa. This mass reduction could be mainly attributed to water evaporation and intensified disruption of the dense, lignified tissue structure. SEM images (Figure 1) revealed that, with increasing pressure and treatment time, the epidermal integrity of the stems was compromised. Cracks, damage and even detachment of the surface layer were observed, exposing the underlying fibrous structure. Under the conditions of 1.4 MPa and 10 min, almost the entire fibrous structure was exposed, with some fibers beginning to be separated. Consequently, the surface of the stems exhibited more prominent protrusions, depressions and wrinkles, resulting in a significant increase in surface roughness.

#### 2.1.2. FT-IR

**Figure 2:**
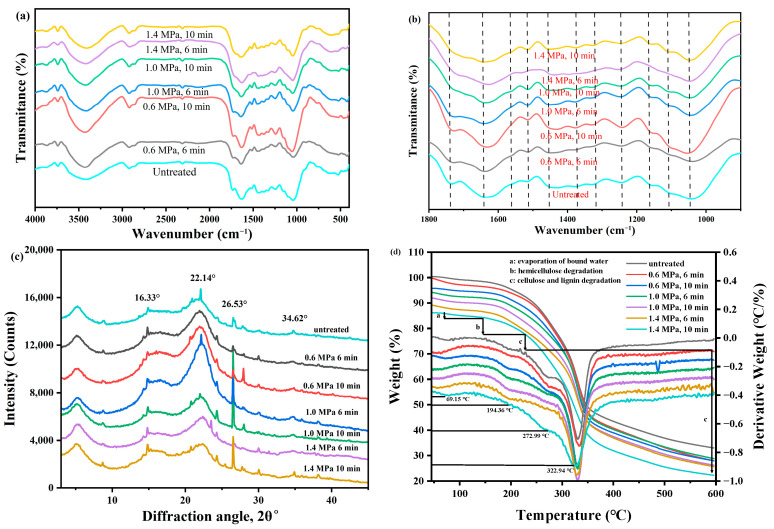
FTIR spectra (**a**,**b**), XRD (**c**) and TGA curves (**d**) of PDB stems under different SE conditions.

According to the IR spectra (Figure 2a,b), the peaks at 3864 cm^−1^, 3735 cm^−1^ and 3412 cm^−1^ were, respectively, attributed to the vibrations of OH groups in lignin, cellulose, and hemicellulose [21]. The peaks at 2918 cm^−1^ and 2855 cm^−1^ corresponded to the C-O bond vibrations [13], while the peaks at 2380 cm^−1^, 2308 cm^−1^ and 1733 cm^−1^ could be attributed to the C=O bond vibrations of carboxyl and acetyl groups in hemicellulose [22]. The peaks at 1636 cm^−1^, 1512 cm^−1^ and 1449 cm^−1^ were due to the C=C skeleton vibrations of aromatic compounds in lignin [23]. The peak at 1376 cm^−1^ was caused by the angular deformation of the C-H group in cellulose [24]. The peak at 1246 cm^−1^ was assigned to the C-O bond of acetyl groups in lignin and ester groups in hemicellulose [13,25]. The peaks at 1155 cm^−1^ and 896 cm^−1^ were attributed to the β-1,4-glycosidic bonds of glucose in cellulose [22,26], and the peak around 1044 cm^−1^ was due to the stretching of the C-O-C bonds in the glucan and xylan rings of cellulose [27,28]. The decreased intensity of the peaks at 1733 cm^−1^, 1636 cm^−1^ and 1449 cm^−1^ indicated a reduction in the corresponding bonds, suggesting a decrease in the content of hemicellulose and lignin. In addition, the peak intensities at 1376 cm^−1^ 1155 cm^−1^, and 896 cm^−1^ showed no significant change, indicating that the cellulose structure remained intact.

The plant cell wall is primarily composed of cellulose and hemicellulose, with lignin filling the spaces between them, forming a robust structure that provides a high hardness and rigidity to the cell wall. The presence of lignin and hemicellulose hinders the penetration of solvents into the other components within plant cells. SE pretreatment technology can reduce the content of hemicellulose and lignin and disrupt the cell wall structure, which facilitates the smooth penetration of solvents into the cells, making it easy to extract active compounds such as phenolics.

#### 2.1.3. XRD Analysis

As shown in Figure 2c, four peaks at 16.33°, 22.14°, 26.53°, and 34.62° corresponded to the characteristic diffraction peaks of cellulose I, which were attributed to the (101), (002), (004), and (040) crystal planes of cellulose [29]. The width and intensity of these peaks reflected the relative crystallinity of samples. Narrow and intense peaks indicated a high crystallinity. The peak at 34.62° became lower and less sharp, suggesting the removal of bonds between the amorphous components (such as hemicellulose and lignin) [22]. Additionally, new crystallization peaks appeared at 28.03°, 30.08°, 35.9°, and 38.16°, indicating an improvement in cellulose crystallinity [30]. From the figure, it could be observed that after SE treatment, the crystalline region of PDB stems relatively increased to the amorphous region. This may be due to the easier degradation of amorphous substances during the SE process [31].

#### 2.1.4. TGA and Derivative Thermogravimetry (DTG) Analysis

The thermal behavior of PDB stems before and after SE pretreatment were also analyzed at a heating rate of 10 °C/min (45–600 °C) under N_2_ atmosphere. From Figure 2d, it could be observed that mass loss initially occurred between 50 and 100 °C, which was primarily because of the loss of bound water in PDB stems [32]. The mass loss between 150 and 250 °C related to the degradation of hemicellulose [31], while the mass loss around 270 °C indicated the degradation of cellulose [31,32]. The degradation at 330 °C provided the evidence for the presence of lignin [24]. It was evident that with increasing SE pressure and duration time, the degradation temperature decreased. In the low-pressure group (0.6 MPa, 10 min), the degradation rate was faster than the untreated stems, likely due to the formation of more pores and defects in the stems caused by the SE process. These structural changes facilitated the rapid mass variation during thermal degradation, resulting in a faster degradation rate. In contrast, the high-pressure group (1.4 MPa, 10 min) may lead to excessive damage to the stems, weakening the structural characteristics responsible for significant mass changes. As a result, the DTG curve showed a lower peak height. However, the basic structural framework of the stems remained intact even after SE pretreatment.

### 2.2. Extraction of Total Phenolics from the SE Treated PDB Stems

Although SE, in theory, can disrupt plant cell walls of PDB stems and enhance the accessibility of intracellular compounds, it needs to be verified through some marked substances like total phenolics. Furthermore, the efficiency of recovery of total phenolics may also significantly influenced by extraction parameters such as ethanol concentration, solid liquid ratio, extraction time, temperature, and the extraction cycles. To gain a more comprehensive understanding of the release behavior of phenolics from PDB following SE pretreatment, this study systematically investigated the effects of various extraction conditions on the yield of total phenolics to optimize the extraction process and provide theoretical support for the efficient utilization of bioactive components in steam-exploded plant materials.

#### 2.2.1. Effect of Ethanol Concentration on Total Phenolics Extraction

As reported in previous studies, ethanol water mixtures are effective for extracting a wide range of phenolic compounds while maintaining their stability [33,34,35]. Typically, ethanol water mixtures are selected as extraction solvents. Given the significant differences in polarity among phenolics, variations in ethanol concentration can markedly affect extraction efficiency. An appropriate ethanol ratio provides a balance between dissolving highly polar phenolics and weakly polar phenolics, thereby improving extraction performance. In this study, the solid-to-liquid ratio was fixed at 1:20 (g/mL), with an extraction time of 60 min, temperature of 45 °C, and one extraction cycle. Under these conditions, ethanol water mixtures with different ethanol concentrations (10%, 30%, 50%, 70%, and 90%, *v*/*v*) were designed to systematically investigate their effects on the extraction efficiency of phenolics. As shown in Table 1, the total phenolic content (mg/g) under different SE conditions and ethanol concentrations were as follows. For these SE treated groups, the extraction efficiencies initially increased with rising ethanol concentration and peaked at 50%, after which it declined. This trend may be attributed to the balanced polarity of 50% ethanol, which enhances the solubility and diffusion of both polar and weakly polar phenolics in PDB. The suitable ethanol water ratio not only facilitates the release of active components but also enhances the solubility and diffusion of a wide range of phenolics. Therefore, 50% ethanol was identified as the optimal extraction solvent and was used in subsequent optimization experiments. Meanwhile, all of the SE treated groups, the content of total phenolics were as much as 2–3 times than the untreated group which proved the SE usefulness.

#### 2.2.2. Effect of Solid Liquid Ratio on Total Phenolics Extraction

The solid liquid ratio is a key parameter that influences the contact area and diffusion efficiency between solute and solvent during plant extraction. An appropriate ratio not only ensures efficient extraction but also increases the concentration of total phenolics while reducing solvent consumption. In this study, based on the optimal ethanol concentration and keeping other conditions constant, the effect of different solid liquid ratios (1:10, 1:20, 1:30, 1:40, and 1:50 g/mL) on the extraction efficiency of total phenolics was systematically investigated. As shown in Table 2, the data are expressed as mg of total phenolic content per gram of sample (mg/g), thus reflecting extraction efficiency relative to sample mass. As the solid liquid ratio decreased, the total phenolic content in all treatment groups first increased and then decreased, with a maximum at 1:20. The relatively low extraction at 1:10 was likely due to insufficient diffusion and incomplete release of phenolic compounds from the tightly structured plant matrix under limited solvent volume. At higher ratios (such as 1:30–1:50), although the solvent volume increased, the improvement in mass transfer was limited, and the extraction yield expressed on a sample-mass basis decreased, indicating that additional solvent did not further enhance the release of phenolics. Therefore, the solid-to-liquid ratio of 1:20 was selected for further optimization. In addition, all of the SE treated groups were significantly higher than the untreated group which was about 2–3 times and proved the SE was useful for the release of total phenolics.

#### 2.2.3. Effect of Extraction Time Total Phenolics Extraction

Extraction time is one of the key factors affecting the efficiency of bioactive compound extraction from plant materials. An appropriate extraction duration facilitates the full release of target compounds from plant cells into the solvent phase, thereby enhancing the extraction efficiency. Insufficient time may lead to incomplete extraction, while excessive duration increases time costs. Therefore, systematically investigating the effect of extraction time on the yield of total phenolics is of great significance for optimizing the extraction process and improving efficiency. Based on the optimal ethanol concentration, solid-to-liquid ratio, and other fixed parameters, the variation in phenolic yield over a time range of 5 to 180 min was studied, and an extraction kinetic curve was constructed to determine the equilibrium time.

The extraction kinetics of total phenolics was investigated on the basis of optimum ethanol concentration and solid liquid ratio, and the results were, respectively, fitted with pseudo-first-order and pseudo-second-order models. The equations for the two models are given below [36]:

(1) $$\ln\left(Q_{e}-Q_{t}\right)=\ln Q_{e}-K_{1}t$$

(2) $$\frac{t}{Q_{t}}=\frac{t}{Q_{e}}+\frac{1}{K_{2}\times Q_{e}^{2}}$$

where *K*_1_ (min^−1^) and *K*_2_ (g(mg/min)) were pseudo-first-order and pseudo-second-order rate constants, respectively. *Q_t_* (mg/g) and *Q_e_* (mg/g) indicated the amount of total phenolics extracted at time *t* and equilibrium, respectively.

**Figure 3:**
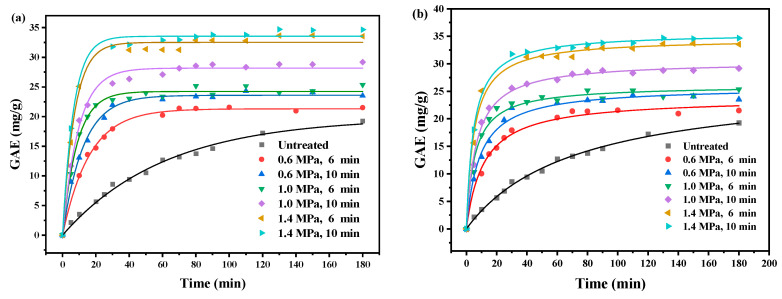

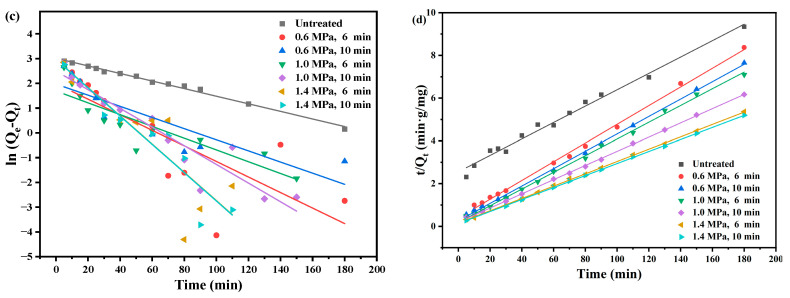
Kinetic modeling of the extraction of total phenolics from the stems of PDB stems: pseudo-first-order model fit curve (**a**), pseudo-second-order model fit curve (**b**), pseudo-first-order model linear fit curve (**c**) and pseudo-second-order model linear fit curve (**d**).

As shown in Figure 3a–d, total phenolic content extracted from SE-treated PDB increased significantly up to about 30 min, which it stabilized and approached equilibrium since 50 min. In contrast, the untreated group did not reach equilibrium even after 180 min. This indicated that SE pretreatment enhanced the extraction efficiency of total phenolics. At 30 min, the extraction amount from the 1.4 MPa, 10 min group was 32.94 mg/g, approximately four times higher than the extraction amount from the untreated group (8.58 mg/g). The high total phenolic content of the SE-treated PDB may be attributed to the reduction in hemicellulose and lignin, which facilitated the smoother penetration of the solvent into the cell interior, making it easier to extract phenolic acids. This finding was consistent with the results from the IR analysis. Therefore, 50 min, the time point at which equilibrium was first reached, was selected for further optimization.

The extraction process was analyzed using both the pseudo-first-order and pseudo-second-order models, with the fitted curves shown in Figure 3a–d. Table 3 presented the quantitative results of both models, including the rate constants (k), total phenolic content (Q_m_), and correlation coefficients (R^2^) for PDB stems under different SE pretreatment conditions. It was observed that the untreated group followed the pseudo-first-order model more closely (0.9914 > 0.9864), which aligned with the previous literature [37]. In contrast, the SE-pretreated PDB stems were better described by the pseudo-second-order model [36], with R^2^ values exceeding 0.99 in all cases.

#### 2.2.4. Effect of Temperature on Total Phenolics Extraction

Temperature is another important parameter affecting the extraction of thermally sensitive compounds such as total phenolics. Therefore, based on the optimal ethanol concentration, solid-to-liquid ratio and extraction time, this study systematically explored the effect of different temperatures (25 °C, 35 °C, 45 °C, 55 °C, and 65 °C) on the extraction efficiency of total phenolics. The results are shown in Table 4 below. As the temperature increased, the extraction efficiencies of total phenolics also increased, reaching the highest value at 65 °C. This could be attributed to the higher temperatures enhanced the solubility of total phenolics in the solvent. Additionally, the increased temperature intensified molecular movement, which boosted the diffusion of total phenolics from the PDB stems into the solvent. This accelerated the time required for the extraction to reach equilibrium, thereby increasing the overall extraction amount. Therefore, 65 °C was selected for further optimization of the extraction cycles.

#### 2.2.5. Effect of Extraction Cycles on Total Phenolics Extraction

Multiple extraction cycles can enhance extraction efficiency and ensure the complete release of target compounds from plant matrices. Moreover, optimizing the cycles of extraction helps to reduce solvent consumption and processing time and improve the economic feasibility of the extraction process. Therefore, determining the optimal extraction cycle is crucial for balancing extraction yield, product integrity, and resource utilization efficiency. In this study (Table 5), the number of extraction cycles (1 to 5) was optimized to achieve maximum extraction efficiency of total phenolics. During the first to third extraction cycles, the total phenolic content increased significantly, indicating that the active components were more readily released at this stage. In contrast, the increases observed during the fourth and fifth extractions were much smaller or nearly negligible, possibly due to the majority of extractable phenolics having already been removed. Therefore, considering economic efficiency, three extraction cycles were selected for subsequent experiments.

### 2.3. Activity of the Extracts from PDB Stems

#### 2.3.1. DPPH Free Radical Scavenging Assay

As shown in Table 6, the IC_50_ values under different SE treated conditions were as follows. The results showed that the IC_50_ values of PDB stems after SE pretreatment were all lower than that of the untreated group, indicating that SE pretreatment facilitated the extraction of active ingredients and enhanced antioxidant activity. The extract of PDB stems under SE pretreatment conditions (0.6 MPa for 10 min) had the lowest IC_50_ value (39.50 ± 0.76 μg/mL), which was a 33.53% decrease to the untreated group (59.43 ± 1.52 μg/mL), demonstrating the strongest antioxidant activity. Compared with the standard control ascorbic acid (IC_50_ = 3.42 ± 0.02 μg/mL), the activity of the PDB stem extract was still lower, but it demonstrated potential as a natural source of bioactive compounds.

#### 2.3.2. ABTS Free Radical Scavenging Assay

As shown in Table 6, the IC_50_ values under different SE conditions were as follows. Compared with the untreated group, the IC_50_ values of PDB stems after SE pretreatment were lower, indicating enhanced ABTS scavenging activity. The extract of PDB stems under 0.6 MPa for 10 min SE pretreatment had the lowest IC_50_ value (61.31 ± 1.31 μg/mL), which was 74.21% of the untreated group (82.6 ± 0.26 μg/mL). Although the activity of the PDB stem extract was lower than that of ascorbic acid (IC_50_ = 20.71 ± 0.42 μg/mL), it confirms that PDB stems can serve as a natural source of bioactive compounds.

#### 2.3.3. FRAP Reduction Assay

As shown in Table 6, at the concentration of 600 μg/mL, the FRAP values were as follows. The FRAP results were consistent with those from the DPPH and ABTS assays. At the same concentration, the absorbance values of SE-pretreated PDB stems were higher than that of the untreated stems. The extract of PDB stems under the 0.6 MPa for 10 min SE pretreatment exhibited the highest absorbance, indicating the strongest ferric reducing antioxidant capacity. At a concentration of 600 μg/mL, the absorbance of the 0.6 MPa, 10 min group (0.7422 ± 0.0029) was 1.56 times higher than that of the untreated group (0.4740 ± 0.0033). Furthermore, as shown in Figure S1, the FRAP results were dose-dependent, similar to ascorbic acid, with absorbance increasing significantly as the sample concentration increased. At a concentration of 600 μg/mL, the absorbance of PDB stems was lower than that of ascorbic acid (3.17 ± 0.05), yet it still highlighted the value of PDB stems as a renewable source of bioactive compounds for further investigation.

#### 2.3.4. α-Glucosidase Inhibition Assay

The hypoglycemic activity of the stems mirrored the antioxidant activity results described above. The IC_50_ values under different SE conditions were as follows. These treated stems exhibited lower IC_50_ values, which demonstrated better hypoglycemic activity compared with the untreated stems. The IC_50_ value of the extract of PDB stems under SE pretreatment (0.6 MPa for 10 min) was 18.51 ± 0.45 μg/mL, representing an 86.16% decrease than that of the untreated stems (133.73 ± 9.60 μg/mL). Compared with the acarbose control (0.00453 ± 0.07 μg/mL), PDB stems exhibited a higher IC_50_ value, yet they could be further explored as a functional ingredient in health-promoting products.

In the SE pretreatment group, as the pressure increased from 0.6 MPa to 1.0 MPa and 1.4 MPa, the IC_50_ values of the extract from PDB stems increased, indicating a decline in both antioxidant and hypoglycemic activities. This reflected that the trends in antioxidant and hypoglycemic activities did not align with the trends in total phenolic content. Higher total phenolic content does not necessarily correlate with higher biological activity [27]. This discrepancy could be attributed to changes in the composition of phenolic compounds, a hypothesis further supported by Q Exactive HF LC-MS analysis below. It is also possible that undetected compounds such as flavonoids, polysaccharides and terpenoids, were affected by the treatment which may not follow the same trend as total phenolic content. These findings indicated that mild treatment conditions were crucial for maintaining bioactivity. Specifically, the samples from SE conditions with lower pressure and longer duration (like 0.6 MPa for 10 min) exhibited the higher antioxidant and hypoglycemic activities. Combined with the optimized extraction parameters (50% ethanol, 1:20 solid-to-liquid ratio, 50 min, 65 °C, three extraction cycles), these mild conditions not only yielded higher total phenolic contents but also maximally preserved the peak areas of bioactive compounds, suggesting that they were not degraded during pretreatment. Therefore, SE treatment under lower pressure and longer duration, together with mild extraction conditions, could avoid excessive damage to cell structures and minimize side reactions, thereby ensuring extraction efficiency while effectively protecting the target bioactive constituents. This provides a reliable sample basis for subsequent functional studies. In addition, mild SE treatment reduces energy consumption, cost, and environmental pollution, while improving product quality.

### 2.4. Identification of the Extracts from PDB Stems

To investigate the reasons for the differences in bioactivity, HPLC analysis was performed on these extracts. As shown in Figure S2, the data revealed significant differences in the chemical composition of PDB stems before and after SE pretreatment. The extracts obtained under the optimal extraction conditions were concentrated by rotary evaporation to remove the solvent, after which the residues from the untreated group and the SE-treated group were separately dissolved in 50% aqueous ethanol and adjusted to the same concentration. Following HPLC analysis, the differences in peak areas at the same compound retention times between the two groups were compared to infer changes in their relative concentrations. This approach provides a more intuitive reflection of the effect of SE pretreatment on the extraction of different components. From the chromatograms, it could be observed that the peak areas of compounds **6**, **8**, **9**, **11**, **12**, **13**, **14** and **18** (corresponding to 3,4-dihydroxybenzaldehyde, 4-hydroxybenzoic acid, catechins, p-coumaric acid, ferulic acid, rutin, rosmarinc acid and ellagic acid) decreased from 4482, 383,305, 45,100, 41,326, 367,592, 892,766, 568,335, 1,857,718 to 3360, 232,365, 19,443, 20,694, 1572, 30,104, 46,043, 6323. The peak areas of compounds **1**, **2**, **3**, **4**, **5**, **7**, **10**, **15**, **16**, and **17** increased (corresponding to gallic acid, hydroquinone, 3,4-dihydroxybenzoic acid, resorcinol, catechol, chlorogenic acid, caffeic acid, luteolin, quercetin and kaempferol) from 172,536, 26,196, 76,973, 1115, 5580, 14,148, 46,524, 10,131, 12,671, 1275 to 4,124,839, 33,896, 233,404, 45,091, 28,762, 23,521, 220,186, 168,376, 185,555, 244,387. These results indicated that SE pretreatment could lead to the degradation of certain compounds or the formation of others, thereby altering their contents and composition, which reflected significant changes in the phytochemical profile. This transformation is likely due to the high temperature and pressure conditions promoting the breakdown of complex macromolecular structures such as lignin and hemicellulose, thereby facilitating the release or formation of small-molecule compounds, particularly phenolic acids and flavonoids. However, the differences in chemical composition among the extracts from different SE treated PDB stems were relatively minor. While slight variations in the content of certain compounds were observed, the overall profiles remained largely consistent. This suggested that once a certain threshold of SE intensity was reached, further increase in treatment severity could not significantly enhance the release of active constituents. One possible explanation is that the main structural disruption and compound transformation occur at the early stage of SE pretreatment, and the additional increases in pressure or duration results in diminishing returns with respect to chemical changes.

**Figure 4:**
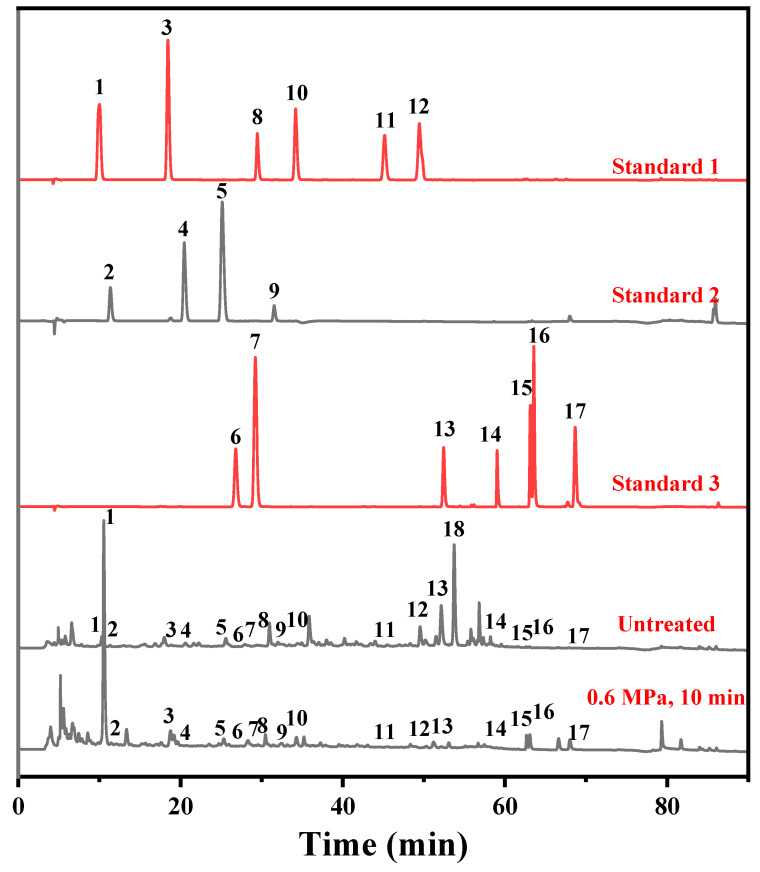
Chromatograms of the extracts from the stems of PDB stems and the standards. 1. gallic acid; 2. hydroquinone; 3. 3,4-dihydroxybenzoic acid; 4. resorcinol; 5. catechol; 6. 3,4-dihydroxybenzaldehyde; 7. chlorogenic acid; 8. 4-hydroxybenzoic acid; 9. catechins; 10. caffeic acid; 11. p-coumaric acid; 12. ferulic acid; 13. rutin; 14. rosmarinc acid; 15. luteolin; 16. quercetin; 17. kaempferol; 18. ellagic acid.

The LC-ESI-MS technique was applied to qualitatively analyze the active constituents of PDB stems, which were rich in phenolic acids, flavonoids, and other compounds according to previous studies and seventeen major active constituents were reasonably deduced by comparing the data with those of the existing studies and standards. In Table S1, compound 1 had deprotonated ion [M-H] at *m*/*z* 169.01402 and a fragment ion at *m*/*z* 125.59845 due to loss of CO_2_, identified as gallic acid [38]. Compound **2**, compound **4**, and compound **5** all had [M-H] ions at *m*/*z* 109.02934. They were identified as hydroquinone, resorcinol, and catechol in turns by comparison of HPLC chromatograms with standards. Compound **3** had a parent ion at *m*/*z* 153.01904 and a fragment ion at *m*/*z* 109.02922 due to loss of CO_2_ and it was identified as 3,4-dihydroxybenzoic acid. The [M-H] ion of compound 6 had fragment ions at *m*/*z* 137.02412 and 121.02927, being identified as 3,4-dihydroxybenzaldehyde [31]. Compound **7** was chlorogenic acid, as it had [M-H] ion at *m*/*z* 353.08661 and fragment ion at *m*/*z* 190.01418 due to a loss of caffeoyl group (C_9_H_8_O_4_) [39]. Compound 8 had [M-H] ions at *m*/*z* 137.02408 and fragment ions at *m*/*z* 108.8996, which was identified as 4-hydroxybenzoic acid. Compound **9**, catechin, had [M-H] ions at *m*/*z* 289.06854 and produced fragment ions at *m*/*z* 125.71696 due to loss of fragments (C_7_H_8_O_2_) [38]. Compound **10** showed [M-H] ion at *m*/*z* 179.03456 and fragment ion at *m*/*z* 135.05029 and was identified as caffeic acid [40]. Compound **11** was designated as p-coumalic acid due to its [M-H] ion at *m*/*z* 163.03970 and fragment ions at *m*/*z* 145.05028 and 117.01908 [41]. Compound **12** was ferulic acid which had [M-H] ion at *m*/*z* 193.05016 and fragment ions at *m*/*z* 175.96828 and 134.04697 [39]. Compound **13** had [M-H] ion at *m*/*z* 609.14220 and formed a fragment ion at *m*/*z* 301.09814 due to the loss of brassinose group (C_6_H_11_O_5_), while the loss of glucose group (C_6_H_10_O_6_) produced a fragment ion at *m*/*z* 271.96259. Thus, it was identified as rutin [42]. Compound **14** was rosemarinic acid, which had [M-H] ions at *m*/*z* 359.07712 with loss of C_7_H_8_O_3_, C_10_H_13_O_4_, and C_11_H_12_O_5_ fragments to form fragment ions at *m*/*z* 258.83151, 180.83954, and 161.84213 [43]. Luteolin was designated as compound **15**, which had [M-H] ions at *m*/*z* 285.03958, loss of CO_2_ resulted in fragmentation ions at *m*/*z* 242.17570, and fragmentation ions at *m*/*z* 151.03966 may be involved in the breakage of the benzene ring and the pyranone ring as well as the genetic rearrangement in the luteolin molecule [44]. Compound **16** had [M-H] ions at *m*/*z* 301.03415 and was identified as quercetin at *m*/*z* 151.03964 due to the formation of fragment ions at a specific positional break in the B-ring of the flavonoid parent nucleus [39]. Compound **17** was kaempferol, which had [M-H] ions at *m*/*z* 285.03976, and was a flavonoid with quercetin, which also formed fragment ions at *m*/*z* 151.00327 due to breakage of the B-ring of the flavonoid parent nucleus at a specific position [45]. As shown in Figure 4, all the above compounds have been compared with the retention times of the corresponding standards by HPLC to verify the correctness of the inference. Compound **18** had [M-H] ions at *m*/*z* 300.99823 and fragment ions at *m*/*z* 256.95474 and was identified as ellagic acid [46].

### 2.5. Compositional Changes in PDB Stems Before and After SE Pretreatment

**Figure 5:**
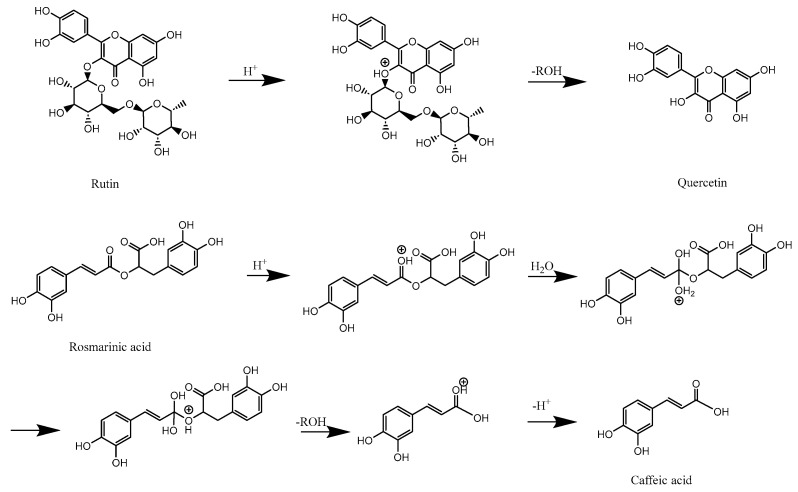
The mechanism of hydrolysis of rutin and rosmarinic acid.

From HPLC results, it was obvious that the substances in PDB stems underwent changes before and after SE pretreatment. Some compounds were degraded, while some new substances were generated. Among these, the contents of rosmarinic acid and rutin showed more significant variations. The variations in rutin (Figure 4, compound **13**), quercetin (Figure 4, compound **16**), rosmarinic acid (Figure 4, compound **14**), caffeic acid (Figure 4, compound **10**), and 4-hydroxybenzoic acid (Figure 4, compound **8**) were compared between the untreated and the 0.6 MPa, 10 min SE-treated groups according to the HPLC chromatograms. Rutin, rosmarinic acid, and 4-hydroxybenzoic acid showed substantial decreases after SE treatment, with their peak areas decreasing from 892,766 to 30,104, 1,857,718 to 46,043, and 383,305 to 232,365, respectively. In contrast, caffeic acid and quercetin exhibited pronounced increases, rising from 46,524 to 220,186 and 12,671 to 185,555, respectively. Rutin content showed a more considerable reduction. This could be attributed to the decrease in the negative logarithm of the ionic product of water (pKw) under high temperature and pressure, where high-pressure steam induced the autohydrolysis of acetyl groups in hemicellulose, leading to the production of acetic acid and the formation of an acidic environment [47]. Additionally, the small amount of water present in the system facilitated the hydrolysis and cleavage of the glycosidic bonds in rutin under high-pressure of SE, high-temperature conditions [48], making it more likely for rutin to hydrolyze into quercetin under acidic conditions [49]. The decrease in rosmarinic acid and the increase in caffeic acid might be due to the hydrolysis of rosmarinic acid into caffeic acid, which aligned with findings from previous studies [31] The hydrolysis mechanisms of rutin and rosmarinic acid under acidic conditions were depicted in Figure 5. This suggested that SE pretreatment led to the decomposition of relatively complex compounds into simple and small molecules, such as monosaccharides and small-molecule phenolic acids. Furthermore, the content of some small molecules, such as 4-hydroxybenzoic acid, also decreased. This reduction could be caused by the large amount of moisture formed during SE, as 4-hydroxybenzoic acid, being water-soluble, might have been carried away by the moisture, resulting in its loss. This process likely enhanced the antioxidant and hypoglycemic activities and increased the bioavailability of PDB stems.

## 3. Materials and Methods

### 3.1. Chemicals and Reagents

2,2-Azinobis-(3-ethylbenthiazoline-6-sulfonate) (ABTS, 98%), potassium hexacyanoferrate (III) (K_3_[Fe(CN)_6_], ≥98%), 3-(trimethoxysilyl) propylme thacrylate (98%), disodium hydrogen phosphate (Na_2_HPO_4_, 99%), chlorogenic acid (≥98%), caffeic acid (98%), rosmarinic acid (98%), ferulic acid (99%), 4-hydroxybenzoic acid (98%), p-coumaric acid (98%), rutin (98%), kaempferol (98%), 3,4-dihydroxybenzaldehyde (98%), 3,4-dihydroxybenzoic acid (98%), and sodium dihydrogen phosphate (NaH_2_PO_4_, 97%) were obtained from Heowns Biochem Technology Co., Ltd. (Tianjin, China). Anhydrous ethanol, phosphoric acid (H_3_PO_4_, 85%) and sodium carbonate (Na_2_CO_3_, 99.8%) were from Tianjin Jiangtian Chemical Technology Co., Ltd. (Tianjin, China). 2,2-Diphenyl-1-picrylhydrazyl (DPPH, ≥98.5%) was from Meryer Chemical Technology Co., Ltd. (Shanghai, China). Acetonitrile (≥99.9%) and methanol (≥99.9%) were from Aladdin Biochemical Technology Co., Ltd. (Shanghai, China). Benzoic acid (C_7_H_6_O_2_, 99.5%) and potassium peroxodisulfate (K_2_S_2_O_8_, ≥99.5%) were from Damao Chemical Reagent Partnership Enterprise (Limited Partnership) (Tianjin, China). Ferric chloride (FeCl_3_, ≥99%) was from Tianjin Yuanli Chemical Co., Ltd. (Tianjin, China). Gallic acid (99%) was from Macklin Biochemical Technology Co., Ltd. (Shanghai, China). α-Glucosidase (≥78.08 U/mg) was from Shanghai Yuanye Biochemical Technology Co., Ltd. (Shanghai, China). p-Nitrophenyl-β-D-glucopyranoside (pNPG, 98%) was from Shanghai Bide Pharmaceutical Technology Co., Ltd. (Shanghai, China). Trichloroacetic acid solution (30%) was from Rionlon (Tianjin, China) Pharmaceutical Co., Ltd. (Tianjin, China). Luteolin (98%), resorcinol (97%), catechol (97%), and quercetin (98%) were from Shanghai Bide Pharmaceutical Technology Co., Ltd. (Shanghai, China). Hydroquinone (98%) was from Fuchen (Tianjin, China) Chemical Reagent Co., Ltd. (Tianjin, China). Resorcinol (99.5%) was purchased from Tianjin Guangfu Fine Chemical Research Institute (Tianjin, China). Catechol (99%) was from Adamas-beta Reagent Co., Ltd. (Shanghai, China). Ultrapure water was from Yongqingyuan Pure Water Manufacturing Center Co., Ltd. (Tianjin, China). The whole herb of PDB was collected in Macheng City, Hubei Province in July 2021 and was identified as PDB by Professor Tianxiang Li of the Tianjin University of Traditional Chinese Medicine. The voucher specimen (No. 202107001) was deposited in the School of Pharmaceutical Science and Technology, Tianjin University, Tianjin.

### 3.2. Instruments

The following instruments were used in the study. PDB stems were pretreated by SE using a QBS-200B device (Hebei Zhengdao Biotechnology Ltd., Shijiazhuang, China). Field emission scanning electron microscope (FEI Company (now part of Thermo Fisher Scientific, (Waltham, MA, USA) was employed to observe the surface microstructure alterations. SmartLab9KW XRD from Rigaku Corporation (Tokyo, Japan) was conducted to investigate the changes in the crystalline structure. TENSOR 27 FT-IR spectrometer from Bruker Corporation (Billerica, MA, USA) was used to characterize PDB stems before and after SE treatment to check the changes in those functional groups. NETZSCH 449f3 TGA from NETZSCH Scientific Instruments Trading (Shanghai, China) was used to analyze the thermal behavior of PDB stems before and after SE. UV-Vis spectrophotometer (Alpha-1506 Shanghai Poyuan Instrument Co., Ltd., Shanghai, China) was employed to measure the absorbance of total phenolics under different extraction conditions. High-performance liquid chromatography (HPLC) system (Chuangxintongheng LC3000, Beijing, China) was used to analyze the compositional changes in the extracts before and after SE treatment. Q Exactive HF LC-MS (Thermo Fisher Scientific, Waltham, MA, USA) was used to identify the total phenolics in the extraction solutions of PDB stems.

### 3.3. Pretreatment of PDB Stems Using SE

PDB stems were selected out one by one and cut into 1 to 2 cm lengths. These stems were divided and each weighing about 200 g was rehydrated at a 1:1 (*w*/*w*) ratio, and they were then sealed in self-sealing bags overnight before SE. The SE process was conducted using a QBS-200B device. In detail, the wetted PDB stems was loaded into the cavity and treated at saturated steam pressures of 0.6 MPa, 1.0 Mpa, or 1.4 MPa for 6 min or 10 min, respectively. Then, the discharge valve was opened to rapidly release the instantaneous pressure and induce the subsequent burst of PDB stems. Following these, the treated PDB stems were immediately collected and dried in an oven at 60 °C until constant weight.

### 3.4. Extraction of Total Phenolics from PDB Stems Before and After SE Pretreatment

Each of the dried sample (1.0 g) was mixed with 50% aqueous ethanol at a solid liquid ratio of 1:20 (g/mL), and extracted at 65 °C in a thermostatic water bath shaker for 60 min. This step was repeated five times. After extraction, the above mixture was centrifuged at 4100 rpm for 10 min, and the supernatant was collected. The total phenolic content (mainly polyphenols) was determined using the Folin Ciocalteu method [50]. The detail procedure was as follows. A 30 μg/mL gallic acid standard solution was used to prepare a series of calibration solutions by transferring 1, 2, 3, 4, or 5 mL into a volumetric flask and diluting each to 5 mL with distilled water. The final concentrations were 6, 12, 18, 24, and 30 μg/mL, respectively. For color development, 1 mL of each standard solution was mixed with 0.5 mL of Folin Ciocalteu reagent, 1 mL of 12% (*w*/*v*) sodium carbonate solution and 2.5 mL of distilled water. The solutions were thoroughly mixed and incubated in a 45 °C water bath for 45 min. Absorbance was then measured at 765 nm using a UV-Vis spectrophotometer, and a standard calibration curve was constructed. For sample analysis, the procedures were the same as above section, and the total phenolic content was calculated according to the standard curve and expressed as gallic acid equivalents (GAE, mg/g).

### 3.5. Activity Evaluation of the Extracts from PDB Stems Before and After SE

To evaluate the bioactivities of PDB stems, DPPH radical scavenging, ABTS radical scavenging, and ferric reducing antioxidant power (FRAP) assays were employed to assess antioxidant activities [51], while α-glucosidase inhibition assay was used to evaluate hypoglycemic activity [52]. These bioactivity assays were conducted only using the extract obtained under the optimized extraction conditions.

#### 3.5.1. DPPH Free Radical Scavenging Assay

DPPH ethanol solution (0.12 μmol/mL, 0.1 mL) was mixed with 0.1 mL different concentrations of PDB stem solutions (10, 25, 50, 75, and 100 μg/mL). Incubation was carried out in a dark environment at 25 °C for 30 min. For standard curve construction, ascorbic acid solutions at concentrations of 2.0, 3.0, 4.0, 7.5, and 9.0 μg/mL were treated in the same manner. The absorbance values were measured at 517 nm using an enzyme meter. All experiments were performed in triplicate, and results are expressed as mean ± standard deviation (SD). The calculation formula was as follows [52].

(3) $$Radical\;scavenging\;rate\;\left(\%\right)=\left(\frac{A_{c}-A_{s}}{A_{s}}\right)\times 100\%$$

where *A_c_* was the absorbance of blank group; *A_s_* was the absorbance of PDB stem group, respectively.

#### 3.5.2. ABTS Free Radical Scavenging Assay

The ABTS radical working solution was prepared by mixing 7 μmol/mL ABTS with 2.45 mmol/L potassium persulfate (K_2_S_2_O_8_) in equal volumes, followed by incubation in the dark at room temperature (about 25 °C) for 12 h. The solution was then diluted fivefold before use. Subsequently, 0.1 mL of sample solutions at different concentrations (10, 25, 50, 75, and 100 μg/mL) were mixed with 0.1 mL of the ABTS working solution and incubated in the dark for 30 min. For standard curve construction, ascorbic acid solutions at concentrations of 2, 4, 8, 15, 25, and 50 μg/mL were treated in the same manner. All experiments were performed in triplicate, and results were expressed as mean ± SD. The absorbance values were measured at 734 nm, and the scavenging rate was calculated using Equation (1).

#### 3.5.3. FRAP Reduction Assay

Extraction solutions of PDB stems at 0.4, 0.6, 0.8, 1.0, or 1.2 mg/mL (0.1 mL each) were mixed with an equal volume (0.1 mL) of 30 μmol/mL K_3_[Fe(CN)_6_] solution (1:1, *v*/*v*) and incubated at 50 °C for 20 min. Then, 0.2 mL of 10% (*w*/*v*) trichloroacetic acid (TCA) solution was added, and the resulting mixture was defined as solution A. Finally, 0.1 mL of solution A was mixed with 0.1 mL of 3.4 μmol/mL FeCl_3_ solution, and the absorbance of the mixture was measured at 700 nm. For standard curve construction, ascorbic acid solutions at concentrations identical to those of the PDB samples (0.40, 0.60, 0.80, 1.00, and 1.20 mg/mL) were treated in the same manner. All experiments were performed in triplicate, and results are expressed as mean ± SD.

#### 3.5.4. α-Glucosidase Inhibition Assay

Extraction solutions of PDB stems at different concentrations (0.05 mL) and α-glucosidase (0.5 U/mL, 0.05 mL) were mixed and reacted at 37 °C for 15 min. Then, the substrate, pNPG (6.0 mmol/L, 0.05 mL), was added to the above mixture and continued the incubation at 37 °C for 15 min. Finally, the reaction was terminated by addition of Na_2_CO_3_ (0.2 mmol/L, 0.05 mL), and the absorbance values was determined at 405 nm. For standard curve construction, acarbose solutions at concentrations of 0.020, 0.010, 0.005, 0.003, and 0.001 mg/mL were treated in the same manner. All experiments were performed in triplicate, and results are expressed as mean ± SD. The inhibition rate was calculated as Equation (2) [52].

(4) $$\alpha -glucosidase\;inhibition\;rate\;\left(\%\right)=\left[1-\frac{\left(A_{S}-A_{S^{\prime }}\right)}{\left(A_{C}-A_{C^{\prime }}\right)}\right]\times 100\%$$

where *A_S_* was the absorbance of α-glucosidase and sample solution, and *A_C_* was the absorbance of α-glucosidase and blank solution. *A_S′_* was the absorbance of the buffer solution and sample solution, and *A_C′_* was the absorbance of the buffer solution and blank solution.

### 3.6. HPLC Analysis

The extraction solutions of PDB stems before and after the SE pretreatment were determined by a HPLC system. Chromatographic conditions: Acclaim^TM^ C18 column (150 mm × 4.6 mm, 5 μm) from Thermo Fisher Scientific (Waltham, MA, USA) was used, and the mobile phases were acetonitrile (A) and 0.1% formic acid aqueous solution (B) with the gradient elution program as follows: 0–15 min, 5–10% A; 15–45 min, 10–20% A; 45–55 min, 20–40% A; 55–70 min, 40% A; 70–75 min, 40–90% A; 75–90 min, 90% A. The flow rate was 0.5 mL/min, and the column temperature was maintained at room temperature. The HPLC analysis was performed using a UV-Vis detector and the detection wavelength was set at 254 nm. The injection volume was 10 μL. The preparation steps for the untreated and SE-treated samples were as follows. The extracts obtained under the optimal extraction conditions (50% ethanol concentration, material-to-liquid ratio of 1:20, extraction time of 50 min, temperature of 65 °C, and three extraction cycles) were concentrated by rotary evaporation to remove the solvent. The residues from the untreated group and the SE-treated group were then separately dissolved in 50% aqueous ethanol and adjusted to the same concentration. The resulting solutions were filtered through a 0.22 μm organic membrane and used as samples for HPLC analysis. For the standards, each compound was accurately weighed and dissolved in 50% aqueous ethanol to prepare a 1 mg/mL solution. Subsequently, 1 mL of each standard solution was mixed to obtain a mixed standard solution, which was then filtered through a 0.22 μm organic membrane for HPLC analysis.

### 3.7. Identification of Total Phenolics in PDB Stems

Q Exactive HF LC-MS was used to identify the total phenolics in the extraction solutions of PDB stems. The untreated group and the SE treated group with the strongest activity were selected for further identification. The chromatographic conditions were the same as Section 3.6. The mass spectrometry was performed using an electrospray ionization (ESI) source in negative ion mode with a capillary voltage of 3.5 kV. Full MS scans were acquired over an *m*/*z* range of 50–1500 with a resolution of 60,000 at *m*/*z* 200. The retention times of the standards and the fragment ions of the mass spectra were compared to confirm the types and structures of total phenolics in the samples. To identify the components in the extract of PDB, its chromatographic profile was compared with those standards using HPLC.

### 3.8. Statistical Analysis

The half maximal inhibitory concentration (IC_50_) was calculated by GraphPad Prism 8.0.2. All data were represented as mean ± standard deviation (SD) and the significant statistical differences was determined by a one-way ANOVA test using SPSS version 22.0. Different lowercase letters were regarded as significant differences (*p* < 0.05).

## 4. Conclusions

SE pretreatment significantly increased the porosity of PDB stems while reducing hemicellulose and lignin contents, with the fundamental structural framework remaining intact. These structural changes facilitated the release of bioactive compounds, particularly total phenolics, resulting in an improved extraction efficiency of 69.14 ± 0.02 mg/g. Bioactivity assays showed that antioxidant activities (DPPH, ABTS, FRAP) and α-glucosidase inhibitory activity were markedly enhanced after SE treatment. Under the condition of 0.6 MPa for 10 min, the activities reached their highest levels, with IC_50_ values of 39.50 ± 0.76, 61.31 ± 1.31, and 18.51 ± 0.45 μg/mL for DPPH, ABTS, and α-glucosidase inhibition, respectively. In the FRAP assay, for example, the absorbance reached 0.7422 ± 0.0029 at a concentration of 600 μg/mL. These results indicated that 0.6 MPa for 10 min could be regarded as the “breakpoint” of mild treatment, at which the target bioactivities were maximally preserved, whereas the higher pressure or prolonged treatment led to activity loss. Furthermore, compositional analysis suggested that some complex structured compounds were partially hydrolyzed into smaller molecules, which may improve the bioavailability of the extracts. In conclusion, the optimized mild SE conditions provided an effective approach for extracting bioactive constituents from PDB stems. A limitation of this study was that bioactivity assays were only conducted under the optimal condition, and further investigations were needed to evaluate different SE parameters and in vivo effects.

## Figures and Tables

**Table 1:** Effect of ethanol concentration on the total phenolics extraction from the SE treated PDB stems.

Conditions	Total Phenolic Contents (mg/g)				
	10%	30%	50%	70%	90%
Untreated	7.59 ± 0.01 ^e^	9.29 ± 0.02 ^c^	11.09 ± 0.01 ^a^	9.44 ± 0.05 ^b^	7.70 ± 0.01 ^d^
0.6 MPa, 6 min	11.34 ± 0.03 ^d^	21.27 ± 0.14 ^b^	22.57 ± 0.01 ^a^	21.04 ± 0.02 ^c^	10.67 ± 0.01 ^e^
0.6 MPa, 10 min	11.45 ± 0.01 ^e^	20.88 ± 0.01 ^b^	24.04 ± 0.10 ^a^	19.45 ± 0.04 ^c^	11.90 ± 0.04 ^d^
1.0 MPa, 6 min	12.07 ± 0.02 ^e^	26.19 ± 0.02 ^b^	27.99 ± 0.01 ^a^	24.68 ± 0.02 ^c^	19.57 ± 0.01 ^d^
1.0 MPa, 10 min	15.35 ± 0.02 ^e^	26.99 ± 0.01 ^b^	28.59 ± 0.03 ^a^	26.40 ± 0.02 ^c^	21.85 ± 0.01 ^d^
1.4 MPa, 6 min	11.72 ± 0.08 ^e^	29.15 ± 0.01 ^c^	33.08 ± 0.05 ^a^	30.06 ± 0.01 ^b^	23.21 ± 0.02 ^d^
1.4 MPa, 10 min	15.30 ± 0.02 ^e^	20.51 ± 0.04 ^c^	36.48 ± 0.02 ^a^	29.38 ± 0.01 ^b^	19.06 ± 0.01 ^d^

Note: ^a–e^ Different superscript letters within the same row indicate statistical differences in total phenol content (*p* < 0.05, one-way ANOVA).

**Table 2:** Effect of solid liquid ratio on the total phenolics extraction from the SE treated PDB stems.

Conditions	Total Phenolic Contents (mg/g)				
	1:10	1:20	1:30	1:40	1:50
Untreated	8.95 ± 0.01 ^c^	11.09 ± 0.01 ^a^	9.35 ± 0.01 ^b^	8.71 ± 0.05 ^d^	6.99 ± 0.01 ^e^
0.6 MPa, 6 min	18.55 ± 0.03 ^c^	22.57 ± 0.01 ^a^	20.66 ± 0.02 ^b^	15.13 ± 0.02 ^d^	12.18 ± 0.01 ^e^
0.6 MPa, 10 min	19.02 ± 0.01 ^c^	24.04 ± 0.10 ^a^	19.70 ± 0.02 ^b^	18.67 ± 0.01 ^d^	10.21 ± 0.01 ^e^
1.0 MPa, 6 min	18.35 ± 0.07 ^c^	27.99 ± 0.01 ^a^	23.68 ± 0.02 ^b^	11.02 ± 0.01 ^d^	10.17 ± 0.02 ^e^
1.0 MPa, 10 min	18.84 ± 0.17 ^b^	28.59 ± 0.03 ^a^	16.83 ± 0.01 ^c^	15.28 ± 0.01 ^d^	11.81 ± 0.01 ^e^
1.4 MPa, 6 min	27.32 ± 0.06 ^c^	33.08 ± 0.05 ^a^	30.41 ± 0.04 ^b^	24.64 ± 0.01 ^d^	17.19 ± 0.01 ^e^
1.4 MPa, 10 min	30.20 ± 0.02 ^b^	36.48 ± 0.02 ^a^	24.99 ± 0.01 ^c^	22.19 ± 0.04 ^d^	15.25 ± 0.01 ^e^

Note: ^a–e^ Different superscript letters within the same row indicate statistical differences in total phenol content (*p* < 0.05, one-way ANOVA).

**Table 3:** Extraction of kinetic model parameters of total phenolics PDB stems.

Conditions	Pseudo-First-Order			Pseudo-Second-Order		
	k1 (/min)	Qm (mg/g)	R^2^	k1 (/min)	Qm (mg/g)	R^2^
Untreated	0.0153	20.3070	0.9914	0.0383	26.0756	0.9864
0.6 MPa, 6 min	0.0314	7.2982	0.5792	0.0437	22.8467	0.9971
0.6 MPa, 10 min	0.0225	7.1919	0.7154	0.0406	24.5942	0.9988
1.0 MPa, 6 min	0.0237	5.4174	0.7574	0.0390	25.5819	0.9980
1.0 MPa, 10 min	0.0377	12.1585	0.8812	0.0332	30.1204	0.9996
1.4 MPa, 6 min	0.0570	19.4638	0.6693	0.0288	34.6740	0.9994
1.4 MPa, 10 min	0.0571	19.3919	0.8459	0.0281	35.5871	0.9997

**Table 4:** Effect of temperature on the total phenolics extraction from the SE treated PDB stems.

Conditions	Total Phenolic Contents (mg/g)				
	25 °C	35 °C	45 °C	55 °C	65 °C
Untreated	5.54 ± 0.02 ^e^	9.12 ± 0.01 ^d^	13.68 ± 0.02 ^c^	19.41 ± 0.01 ^b^	23.93 ± 0.03 ^a^
0.6 MPa, 6 min	12.28 ± 0.002 ^e^	14.41 ± 0.02 ^d^	17.70 ± 0.04 ^c^	21.64 ± 0.01 ^b^	27.06 ± 0.08 ^a^
0.6 MPa, 10 min	10.50 ± 0.01 ^e^	17.57 ± 0.03 ^d^	20.09 ± 0.05 ^c^	22.17 ± 0.06 ^b^	31.41 ± 0.06 ^a^
1.0 MPa, 6 min	15.95 ± 0.01 ^e^	20.06 ± 0.07 ^d^	24.10 ± 0.03 ^c^	30.59 ± 0.04 ^b^	39.54 ± 0.03 ^a^
1.0 MPa, 10 min	15.92 ± 0.03 ^e^	16.75 ± 0.01 ^d^	28.36 ± 0.04 ^c^	29.26 ± 0.07 ^b^	39.83 ± 0.08 ^a^
1.4 MPa, 6 min	21.32 ± 0.04 ^e^	23.97 ± 0.01 ^d^	31.16 ± 0.02 ^c^	34.09 ± 0.02 ^b^	42.61 ± 0.02 ^a^
1.4 MPa, 10 min	14.24 ± 0.01 ^e^	23.89 ± 0.02 ^d^	27.75 ± 0.003 ^c^	38.62 ± 0.08 ^b^	38.91 ± 0.05 ^a^

Note: ^a–e^ Different superscript letters within the same row indicate statistical differences in total phenol content (*p* < 0.05, one-way ANOVA).

**Table 5:** Effect of extraction cycles on the total phenolics extraction from the SE treated PDB stems.

Conditions	Total Phenolic Contents (mg/g)				
	1	2	3	4	5
Untreated	18.76 ± 0.04 ^e^	30.54 ± 0.04 ^d^	34.54 ± 0.03 ^c^	35.95 ± 0.12 ^b^	36.95 ± 0.02 ^a^
0.6 MPa, 6 min	25.46 ± 0.04 ^e^	35.06 ± 0.06 ^d^	41.07 ± 0.01 ^c^	39.55 ± 0.04 ^b^	44.46 ± 0.06 ^a^
0.6 MPa, 10 min	26.35 ± 0.02 ^e^	35.18 ± 0.02 ^d^	38.06 ± 0.09 ^c^	38.77 ± 0.02 ^b^	39.58 ± 0.03 ^a^
1.0 MPa, 6 min	27.40 ± 0.07 ^e^	37.51 ± 0.02 ^d^	42.23 ± 0.02 ^c^	44.73 ± 0.03 ^b^	48.04 ± 0.01 ^a^
1.0 MPa, 10 min	29.51 ± 0.03 ^e^	41.24 ± 0.01 ^d^	46.37 ± 0.01 ^c^	45.44 ± 0.07 ^b^	50.98 ± 0.30 ^a^
1.4 MPa, 6 min	33.22 ± 0.07 ^e^	47.81 ± 0.04 ^d^	47.78 ± 0.02 ^c^	50.19 ± 0.04 ^b^	53.65 ± 0.04 ^a^
1.4 MPa, 10 min	39.07 ± 0.06 ^e^	52.88 ± 0.07 ^d^	69.14 ± 0.02 ^c^	68.90 ± 0.11 ^b^	67.60 ± 0.04 ^a^

Note: ^a–e^ Different superscript letters within the same row indicate statistical differences in total phenol content (*p* < 0.05, one-way ANOVA).

**Table 6:** In vitro antioxidant and hypoglycemic activities of PDB stems with different SE pretreatment conditions.

Sample	DPPH·IC_50_ (μg/mL)	ABTS·IC_50_ (μg/mL)	FRAP (A_700_, 600 μg/mL)	α-Glucosidase IC_50_ (μg/mL)
Ascorbic acid	3.47 ± 0.02 ^a^	20.71 ± 0.42 ^a^	3.17 ± 0.05 ^a^	-
Acarbose	-	-	-	0.00453 ± 0.07 ^a^
Untreated	59.43 ± 1.52 ^f^	82.6 ± 0.26 ^e^	0.4740 ± 0.0033 ^g^	133.73 ± 9.60 ^f^
0.6 MPa, 6 min	42.05 ± 0.32 ^c^	61.85 ± 1.17 ^b^	0.7419 ± 0.0042 ^b^	32.55 ± 3.77 ^c^
0.6 MPa, 10 min	39.50 ± 0.76 ^d^	61.31 ± 1.31 ^b^	0.7422 ± 0.0029 ^c^	18.51 ± 0.45 ^b^
1.0 MPa, 6 min	45.82 ± 0.49 ^d^	71.16 ± 0.61 ^c^	0.6662 ± 0.0035 ^d^	62.96 ± 9.26 ^d^
1.0 MPa, 10 min	47.61 ± 1.45 ^de^	72.61 ± 0.41 ^c^	0.6561 ± 0.0016 ^e^	69.34 ± 7.81 ^d^
1.4 MPa, 6 min	48.88 ± 1.09 ^e^	75.42 ± 0.95 ^d^	0.6080 ± 0.0015 ^e^	71.70 ± 4.46 ^d^
1.4 MPa, 10 min	48.92 ± 1.80 ^f^	75.22 ± 0.79 ^e^	0.6045 ± 0.0034 ^f^	89.67 ± 9.57 ^f^

Note: ^a–g^ Different superscript letters in the same column indicate statistically significant differences in IC_50_ or absorbance (*p* < 0.05, one-way ANOVA).

## Data Availability

All data are available from the authors.

## Supplementary Materials

The following supporting information can be downloaded at: https://www.mdpi.com/article/10.3390/molecules31010139/s1, Figure S1: FRAP results of the extracts from the different SE treated PDB stems; Figure S2: HPLC chromatograms of the extracts from the stems of PDB under different SE treated conditions; Table S1: LC-UV-MS/MS information of compounds [31,34,38,40,41,42,43,44,45].

## Abbreviations

The following abbreviations are used in this manuscript:

SE	Steam explosion
SEM	Scanning electron microscopy
TGA	Thermogravimetric analysis
XRD	X-ray diffraction
FTIR	Fourier transform infrared spectroscopy
HPLC	High performance liquid chromatography
Q Exactive HF LC-MS	Quadrupole-Orbitrap exactive high field liquid chromatography-mass spectrometry
