# Supplementary material for: Steam Explosion as a Green Pretreatment Strategy to Enhance Total Phenolics Release and Biological Activities in Potentilla discolor Bunge Stems
Source: Molecules. 2025 Dec 31;31(1):139. doi: 10.3390/molecules31010139 (PMC12787479; doi:10.3390/molecules31010139)
Supplement: Supplementary file 1 [file molecules-31-00139-s001.zip › molecules-4007714-supplementary.pdf]

Supplementary materials for "Steam explosion as a green pretreatment strategy to enhance total phenolics release and biological activities in *Potentilla discolor* Bunge stems"

Xiao Zhang[1], Yuchen Cui[1], Wenjie Sui[2], Mengqi Cheng[1], Xinyu Xu[1], Wanting Duan[1], Ziyi Cheng[1], Jiajia Fu[1], Yanmei Xu[1,3,*], Youxin Li[1,4,5,*]

1 Tianjin Key Laboratory for Modern Drug Delivery and High-Efficiency, Collaborative Innovation Center of Chemical Science and Engineering, School of Pharmaceutical Science and Technology, Faculty of Medicine, Tianjin University, Tianjin 300072, China.
2 State Key Laboratory of Food Nutrition and Safety, Tianjin University of Science & Technology, Tianjin 300457, China
3 Hebei Institute for Drug and Medical Device Control, Shijiazhuang, Hebei 050033, China
4 Neurocritical Care Medicine Innovation Center, Ministry of Education, Tianjin University, Tianjin 300072, China
5 State Key Laboratory of Advanced Medical Materials and Devices, Tianjin University, Tianjin 300072, China
* Correspondence: lyx@tju.edu.cn (Y.L.); xym123_@tju.edu.cn (Y.X.).

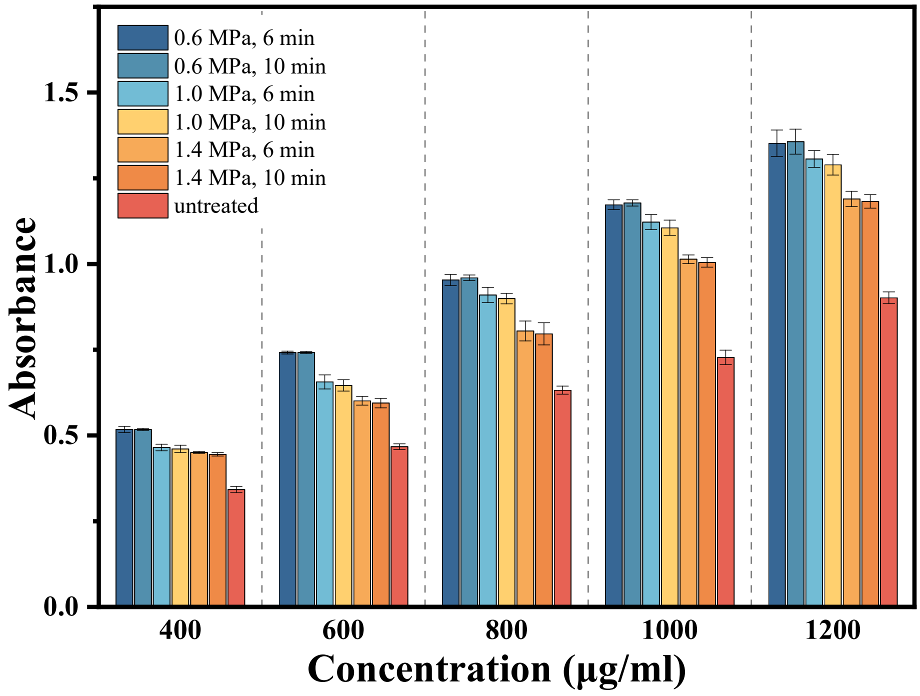

Figure S1. FRAP results of the extracts from the different SE treated PDB stems

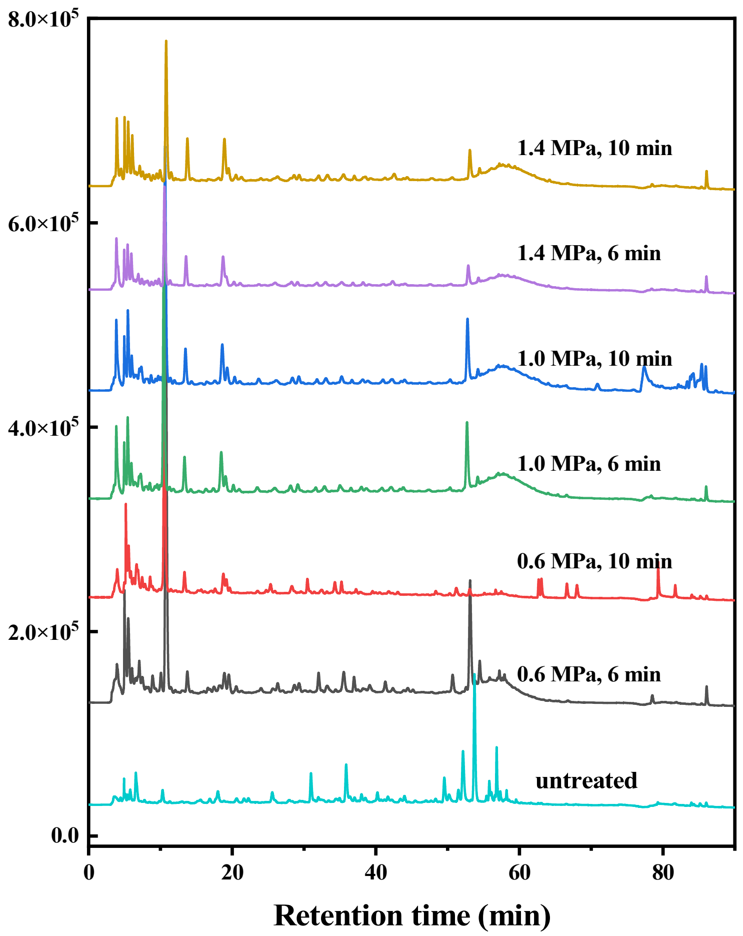

Figure S2. HPLC chromatograms of the extracts from the stems of PDB under different SE treated conditions

Table S1. LC-UV-MS/MS information of compounds

Molecular formula	Theoretical value [m-h]	Actual value [m-h]	Inaccuracy（ppm）	Retention time（min）	Fragmentation ion	Compound	
$C_7H_6O_5$	169.01315	169.01402	5.14928	9.97	125.59845	Gallic acid	[1]
$C_6H_6O_2$	109.02950	109.02934	-1.46749	11.4		hydroquinone	
$C_7H_6O_4$	153.01933	153.01904	-1.90826	18.5	137.02406<br>109.02922	3,4-Dihydroxybenzoic acid	
$C_6H_6O_2$	109.02950	109.02934	-1.46749	20.21		resorcinol	
$C_6H_6O_2$	109.02950	109.02935	-1.37578	24.79		catechol	
$C_7H_6O_3$	137.02332	137.02412	5.83474	26.8	121.02927	3,4-Dihydroxybenzaldehyde	[2]
$C_{16}H_{18}O_9$	353.08671	353.08661	-0.27897	29.37	190.01418	Chlorogenic acid	[3]
$C_7H_6O_3$	137.02332	137.02408	5.54282	29.67	108.8996<br>119.5246	4-hydroxybenzoic acid	
$C_{15}H_{14}O_6$	289.07066	289.06854	-7.34981	31.64	109.07091	Catechins	[1]

					125.71696		
$C_9H_8O_4$	179.03389	179.03456	3.76910	34.06	135.05029	caffeic acid	[4]
$C_9H_8O_3$	163.04006	163.03970	-2.20805	45.4	145.05028<br>117.01908	coumalic acid	[5]
$C_{10}H_{10}O_4$	193.05060	193.05016	-2.27920	49.53	175.96828<br>134.04697	Ferulic acid	[3]
$C_{27}H_{30}O_{16}$	609.14611	609.14220	-6.41541	52.43	448.99155<br>271.96259<br>301.09814	Rutin	[6]
$C_{14}H_6O_8$	302.00681	302.00482	-6.58930	53.70	256.95474	ellagic acid	
$C_{18}H_{16}O_8$	359.07614	359.07712	2.71864	56.68	180.83954<br>161.84213	Rosmarinc acid	[7]
$C_{15}H_{10}O_6$	285.04046	285.03958	-3.09080	62.51	258.83151<br>242.17570<br>151.03966	Luteolin	[8]
$C_{15}H_{10}O_7$	301.03428	301.03415	-0.42885	63.66	178.84238<br>151.03964	Quercetin	[3]
$C_{15}H_9O_6$	285.03936	285.03976	1.38753	68.65	256.03479<br>228.88327<br>151.00327	Kaempferol	[9]

1. Liu, Z.; Bruins, M. E.; Bruijn, W. J. C. D.; Vincken, J. P., A comparison of the phenolic composition of old and young tea leaves reveals a decrease in flavanols and phenolic acids and an increase in flavonols upon tea leaf maturation. *Elsevier BV* **2020**.
2. Wang, J.; Zhang, X.; Li, S.; Zhang, T.; Yang, S.; Chen, H.; Sui, W.; Zhang, M., Physical properties, phenolic profile and antioxidant capacity of Java tea (Clerodendranthus spicatus) stems as affected by steam explosion treatment. *Food Chemistry* **2024**, (May 15), 440.
3. Wang, C.; Zhang, M.; Wu, L.; Wang, F.; Li, L.; Zhang, S.; Sun, B., Qualitative and quantitative analysis of phenolic compounds in blueberries and protective effects on hydrogen peroxide-induced cell injury. *Journal of separation science* **2021,** 44, (14), 2837-2855.
4. Wenyi, L.; Wenjing, C.; Lingfang, W.; Shi, L.; Qi, Q.; Yaping, C.; Linjin, L.; Ting, Y.; Lanzhen, Z., Quality Evaluation and Chemical Markers Screening of Salvia miltiorrhiza Bge. (Danshen) Based on HPLC Fingerprints and HPLC-MSn Coupled with Chemometrics. *Molecules* **2017,** 22, (3), 478.
5. Kolniak-Ostek; Joanna, Identification and quantification of polyphenolic compounds in ten pear cultivars by UPLC-PDA-Q/TOF-MS. *Journal of Food Composition & Analysis* **2016,** 49, 65-77.
6. B, C. L. A.; B, C. Z.; B, Q. N.; A, B. Y.; B, B. Z.; C, S. D. A., Simultaneous determination of seven flavonoids, two phenolic acids and two cholesterines in Tanreqing injection by UHPLC-MS/MS - ScienceDirect. *Journal of Pharmaceutical and Biomedical Analysis* **2019,** 163, 105-112.
7. Guo, Z.; Liang, X.; Xie, Y., Qualitative and quantitative analysis on the chemical constituents

in Orthosiphon stamineus Benth. using ultra high-performance liquid chromatography coupled with electrospray ionization tandem mass spectrometry. *Journal of Pharmaceutical and Biomedical Analysis* **2019,** 164, 135-147.

8. Luo, Y.; Lai, C. J. S.; Zhang, J.; Feng, Y.; Wen, Q.; Tan, T., Comprehensive metabolic profile of phenolic acids and flavonoids in Glechomae Herba using ultra-high-performance liquid chromatography coupled to quadrupole-time-of-flight tandem mass spectrometry with diagnostic ion filtering strategy. *Journal of Pharmaceutical and Biomedical Analysis* **2019,** 164, (000), 15.

9. Yu, C.; Shengyun, L.; Xuemei, Z.; Aijin, M.; Zhixiang, C.; Guohui, Q.; Suping, G.; and tian, Y., Purification and ultra-high-performance liquid chromatography tandem mass spectrometry analysis of phenolics extracted from male walnut flowers. *International Journal of Food Properties* **2022,** 25, (1), 1792-1803.
